# Aberrant STYK1 expression in ovarian cancer tissues and cell lines

**DOI:** 10.1186/1757-2215-2-15

**Published:** 2009-10-21

**Authors:** Kesmic A Jackson, Gabriela Oprea, Jeffrey Handy, K Sean Kimbro

**Affiliations:** 1 Department of Hematology and Medical Oncology, Winship Cancer Institute, Emory University School of Medicine, Building C, Room C4090, Atlanta, GA 30322, USA; 2 Pathology and Laboratory Medicine, Emory University School of Medicine, Atlanta, GA, USA; 3 Division of Digestive Diseases, Emory University School of Medicine, Atlanta, GA, USA

## Abstract

**Background:**

Overexpression of *STYK1*, a putative serine/threonine and tyrosine receptor protein kinase has been shown to confer tumorigenicity and metastatic potential to normal cells injected into nude mice. Mutation of a tyrosine residue in the catalytic STYK1 domain attenuates the tumorigenic potential of tumor cells *in vivo*, collectively, suggesting an oncogenic role for STYK1.

**Methods:**

To investigate the role of STYK1 expression in ovarian cancer, a panel of normal, benign, and ovarian cancer tissues was evaluated for STYK1 immunoreactivity using STYK1 antibodies. In addition, mRNA levels were measured by reverse transcription PCR and real-time PCR of estrogen receptors, GPR30 and STYK1 following treatment of ovarian cell lines with estrogen or G1, a GPR30 agonist, as well as western analysis.

**Results:**

Our data showed higher expression of STYK1 in cancer tissues versus normal or benign. Only normal or benign, and one cancer tissue were STYK1-negative. Moreover, benign and ovarian cancer cell lines expressed *STYK1 *as determined by RT-PCR. Estradiol treatment of these cells resulted in up- and down-regulation of *STYK1 *despite estrogen receptor status; whereas G-1, a GPR30-specific agonist, increased STYK1 mRNA levels higher than that of estradiol.

**Conclusion:**

We conclude that *STYK1 *is expressed in ovarian cancer and is regulated by estrogen through a GPR30 hormone-signaling pathway, to the exclusion of estrogen receptor-alpha.

## Introduction

Ovarian cancer causes more deaths in women than any other gynecological cancer. The number of deaths caused by ovarian cancer is exacerbated by the lack of reliable screening, specific symptoms, and effective treatments. The National Cancer Institute estimates that 21,550 new cases of ovarian cancer will be diagnosed in the US in 2009. Women diagnosed with localized, regional, and distant ovarian cancer have a 93%, 69%, and 30% 5-year survival rate, respectively [1-3]. However, diagnosis of localized ovarian cancer only occurs in about 19% of the cases due to a lack of reliable screening techniques and the absence of specific symptoms.

Ovarian cancer samples overexpress a putative serine-threonine receptor protein kinase, *STYK1*, as demonstrated by microarray analysis [4]. The human STYK1 kinase domain shares approximately 30-34% identity with FGFR (fibroblast growth factor receptor)/PDGFR (platelet-derived growth factor) family members, which have been shown to function as oncogenes [5]. STYK1 overexpression constitutively activated the RAS/MAPK, STAT1, and STAT3 pathways in NIH3T3 cells [6]. Interestingly, ovarian cancer cells were shown to constitutively express high levels of STAT3 [7,8]. Furthermore, BaF3 cell lines overexpressing *STYK1 *proliferated in media without serum or growth factors. Inoculation of these cells into nude mice induced tumor formation within one week and the cells metastasized after 4 weeks. Introducing a tyrosine to phenylalanine point mutation into the catalytic domain of STYK1 blocked cell proliferation as well as STYK1-induced tumorigenesis [6,9]. *STYK1 *expression is regulated by estrogen in ERα (estrogen receptor alpha)-negative (MDA-MB-231) and ERα-positive MCF7) breast cancer cells based on microarray analysis and real-time PCR analysis [10].

Estrogen receptors play a critical role in ovarian tumor cell growth. Ovarian surface epithelial cells produce estradiol and estrone, and the ovary is a key target of estrogen [11]. The postmenopausal ovary produces little or no estrogen; conversely, increased steroid hormone levels have been observed in the plasma of ovarian cancer patients [12]. The occurrence of ovarian cancer increases dramatically in menopausal women. Furthermore, previous studies report a correlation between plasma estradiol, progesterone, and androstenedione with stage of disease [13,14]. However, the mechanisms by which estrogen receptors contribute to ovarian tumorigenesis are still unclear [4]. GPR30, a novel estrogen receptor, and ERα stimulation by both G-1 (GPR30-specific ligand) and estradiol were shown to synergistically induce proliferation of breast and ovarian cancer lines [15].

In this study we examined STYK1 immunoreactivity in normal, benign, and malignant ovarian tissues. To investigate the role of estrogen and GPR30 in STYK1 regulation, we treated a benign and several malignant ovarian cancer cell lines with estradiol and G-1. We describe differences in STYK1 RNA and protein expression levels in treated versus untreated ovarian tumor cells. We also compare estradiol- and G-1-induced STYK1 expression. In the present report, we show that STYK1 expression is associated with ovarian tumorigenesis. Furthermore, we provide evidence for estrogen-mediated *STYK1 *regulation through an unknown GPR30 signaling pathway.

## Materials and methods

### Chemicals

17β-estradiol and BSA-conjugated estradiol were purchased from Sigma-Aldrich (Sigma, St. Louis, MO). 1-(4-(6-Bromobenzo[1,3]dioxol-5-yl)-3a,4,5,9b-tetrahydro-3H-cyclopenta [c]quinolin-8-yl)-ethanone (G-1) was purchased from Calbiochem (San Diego, CA).

### Antibodies

STYK1 and GPR30 antibodies were purchased from AbCam (Cambridge, MA). α-Tubulin antibody was purchased from Millipore (Billerica, MA).

### Cell culture

HS832, OvCar3, and CaOv3 were obtained from American Type Culture Collection (Manassas, VA). SkOv3, OvCar5, OvCar8, and IGROV1 were kindly provided by the lab of Dr. Neil Sidell (Emory University School of Medicine, Department of Gynecology and Obstetrics). All cell lines were maintained in DMEM with 10% FBS. Prior to treatment the cells were incubated in phenol-red free DMEM supplemented with 20% charcoal stripped FBS overnight (12-16 h) followed by incubation with 5 × 10^-8 ^M estradiol, 1 × 10^-8 ^M BSA-conjugated estradiol, and 1 × 10^-8 ^M G-1 for 4-18 h. Ethanol, phosphate-buffered saline (PBS), and dimethyl sulfoxide were used as the respective vehicle controls.

### Reverse transcriptase (RT) and real time RT-PCR

Treated and untreated cells were rinsed with PBS and pelleted for RNA isolation. RNA was extracted using the RNeasy Midi kit (Qiagen Inc., Valencia, CA) according to the manufacturer's instructions. RNA purity and concentration were determined by spectrophotometry. cDNA was generated at a concentration equivalent to 25 ng/μL of RNA in a 20 μL volume with random hexamers and Superscript II reverse transcriptase (Invitrogen Corporation). The PCR products were visualized on ethidium bromide-stained 2% agarose gels under UV light. Real-time PCR was carried out using the ABI Prism 7000 System. Tubulin was used as an internal control for normalization of each data point. Relative induction was calculated using the 2^-ΔΔCT ^formula [16]. RNA was analyzed from three independent experiments.

### Western blotting

Lysates were collected from treated and untreated cells in modified radioimmuno precipitation assay (RIPA) buffer containing EDTA and a protease inhibitor cocktail (Pierce Biotechnology, Rockford, IL) by standard methods. 40 μg of protein was resolved by SDS-PAGE and transferred onto PVDF membranes. The membranes were subjected to immunodetection by incubation with primary antibody for STYK1 (1:500) and GPR30 (1:250). Equal protein loading was controlled by immunoblot of α-tubulin (1:3000). The lysates from three independent experiments were analyzed.

### Tissue panel and immunohistochemistry

Formalin-fixed arrays of normal, benign, and malignant ovarian tissues were obtained from Pantomics Inc. (San Franscico, CA). The tissues were stained with a mouse monoclonal antibody for STYK1 and counterstained with hematoxylin by the Winship Cancer Institute Pathology Core Facility at Emory University. Each tissue section was assigned a score of 0 for none, 1 for weak, 2 for moderate, or 3 for strong STYK1 immunoreactivity. Scoring of the tissue sections was done by one of the authors without prior knowledge of the clinical parameters.

### Statistical analysis

Statistical analyses were performed using the one-way ANOVA test in GraphPad Prism (San Diego, CA). The data are presented as mean ± standard error.

## Results

### Expression of STYK1 in normal, benign, and malignant ovarian tissues

**Figure 1:**
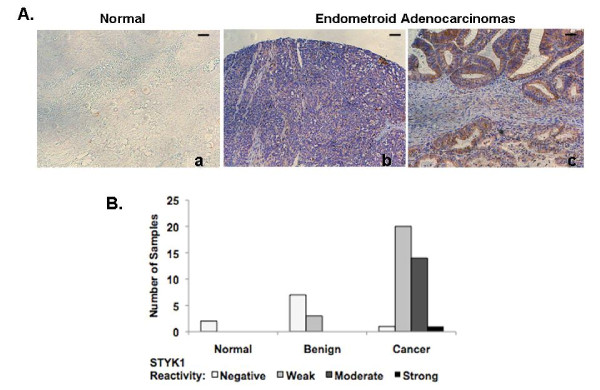
**STYK1 Protein Expression is Associated with Ovarian Cancer**. An ovarian tissue array (Pantomics, Inc.) was stained with STYK1 antibody. (A) Each tissue section was assigned a score of 0 for no staining, 1 for weak, 2 for moderate, or 3 for strong STYK1 reactivity. (B) STYK1 localizes to the cytoplasm in malignant ovarian tissues. Representative of sections (20× magnification) immunohistochemical stains of normal and endometroid adenocarcinomas with anti-STYK1 antibody shown, from left to right, no staining (a), weak (b), and moderate staining (c).

Each normal ovarian tissue section was negative for STYK1 immunoreactivity (Fig. 1A). Although several of the benign ovarian tissue sections were positive for STYK1 immunoreactivity the staining intensity was weak (represented by the staining in normal tissue). The remaining benign ovarian tissues as well as one malignant ovary showed no STYK1 immunoreactivity. Many of the ovarian cancer tissue sections had weak STYK1 staining intensity as seen in the benign tissues, however, moderate and strong STYK1 staining intensity was seen only in the malignant ovarian tissues (i.e. endometroid adenocarcinomas). STYK1 immunoreactivity was cytoplasmic in every STYK1-positive ovarian tissue section (Fig. 1B).

### Expression of estrogen receptors and STYK1 in ovarian cancer cell lines

**Figure 2:**
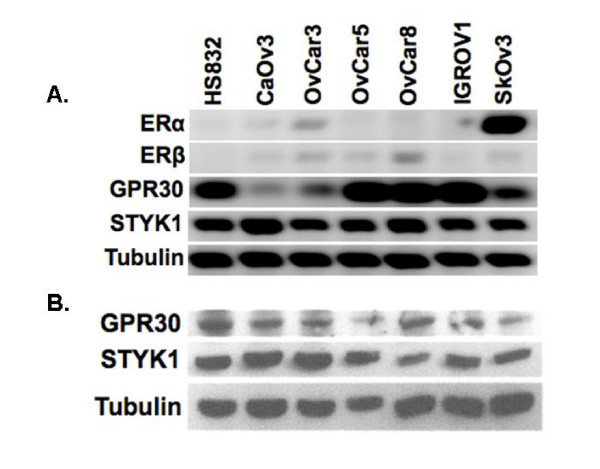
**Ovarian tumor cell lines express STYK1 and variably express estrogen receptors**. HS832 is a benign ovarian cell line and the remaining cell lines were derived from ovarian cancer cell lines. (A) Ethidium bromide-induced fluorescence of RT-PCR amplification product from ovarian tumor RNA using primers for ERα, ERβ, GPR30, and *STYK1*. Tubulin primers were used as a loading control; (B) STYK1 and GPR30 protein expression. The western blot was stripped and re-probed with α-tubulin antibody as a loading control.

We detected ER-*α *RNA expression in ovarian cancer cell lines SKOV3, CaOv3, and OvCar3 (Fig. 2A). Cell lines HS832, OvCar5, OvCar8 and IGROV had no detectable ER*α *transcript. While ER*β *expression was weak for most of the cell lines, no expression was detected in HS832 and IGROV1 cells. Every cell line expressed *GPR30*; however, strong expression was seen only in HS832, OvCar5, OvCar8, and IGROV1. GPR30 and STYK1 protein was detected at varying levels in each cell line (Fig. 2B).

### Estradiol and GPR30-specific G-1 induce STYK1 RNA but not protein expression in ovarian cancer cell lines

**Figure 3:**
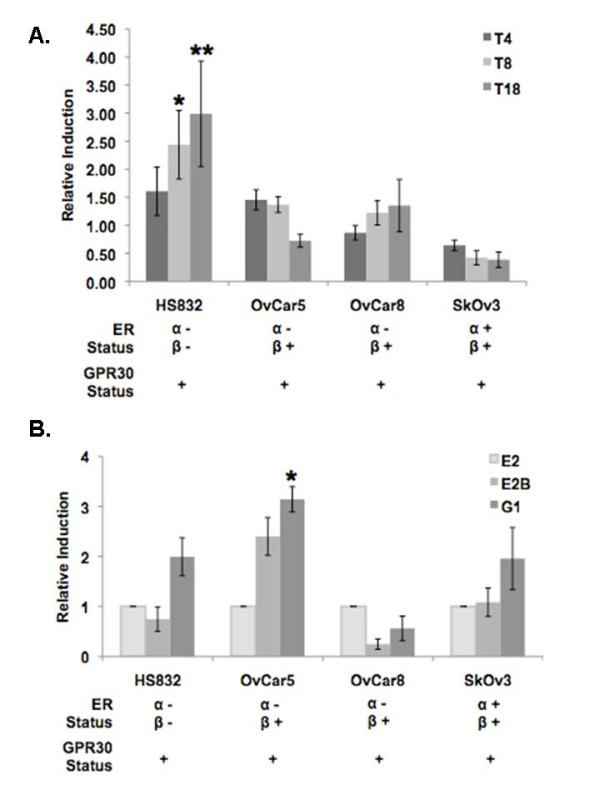
**Estradiol and G-1 induce STYK1 RNA expression**. Ovarian tumor cell lines were treated with vehicle, 5 × 10^-8 ^M estradiol, 1 × 10^-8 ^M BSA-conjugated estradiol (E2B), and 1 × 10^-8 ^M G-1 for 4-18 h(T4, T8, T18). cDNA equivalent to 25 ng/μL of RNA was generated and analyzed by real-time RT-PCR. Relative values were normalized to α-tubulin and values were compared to the vehicle. Values are the mean of 3 independent experiments. (A) STYK1 induction in estradiol-treated ovarian tumor cells relative to untreated cells for various intervals; (B) STYK1 induction in E2-, E2B- and G1-treated cells relative to estradiol-induced STYK1 expression following 18 hours. * and ** indicate values that are significantly different compared to the vehicle (p < 0.01 and p < 0.001, respectively).

RNA isolated from estradiol-treated HS832, OvCar5, OvCar8, and SkOv3 cells was analyzed by real time RT-PCR with *STYK1 *primers. *STYK1 *expression increased significantly (p < 0.001) in HS832 cells (ER*α *neg., *ER-β *neg., GPR30 pos.) and decreased by almost half in the SkOv3 cells (ER*α *pos., *ER-β *pos., GPR30 pos.) after 18 hours in the presence of estradiol (Fig. 3A). While *STYK1 *expression decreased slightly in OvCar5 cells (ER*α *neg., *ER-β *pos., GPR30 pos.), there was no real change in expression in OvCar8 cells, which have the same ER expression profile.

**Figure 4:**
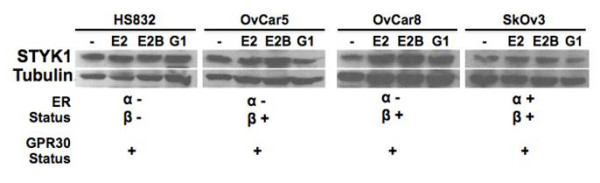
**STYK1 protein expression is unaffected by estradiol and G-1 treatment**. Ovarian tumor cell lines were treated with vehicle, 5 × 10^-8 ^M estradiol, 1 × 10^-8 ^M BSA-conjugated estradiol (E2B), and 1 × 10^-8 ^M G-1 for 4-18 h for 18 hours. Forty μg of protein lysate from the indicated cell lines were electrophoresed by 12.5% SDS PAGE and transferred to PVDF membrane. The blot was probed with anti-STYK1 antibody then stripped and re-probed with α-tubulin as a loading control. This is a representative blot from three independent experiments. * indicates a value that is significantly different compared to 5 × 10^-8 ^M estradiol treatment (p < 0.001).

HS832, OvCar5, and SkOv3 cells treated (16 hours) with G-1, a GPR30-specific ligand, showed an increase in *STYK1 *expression relative to 16 hours estradiol-treated cells although the increase was only significant in OvCar5 cells (p < 0.001; Fig. 3B). Conversely, *STYK1 *expression decreased slightly in G-1-treated OvCar8 cells. When the cells were treated with BSA-conjugated estradiol (E2B), which allows estradiol to interact with receptors on the cell membrane but prevents the molecule from entering the cell, *STYK1 *expression increased relative to estradiol-induced expression in OvCar5 cells but decreased in OvCar8 cells. There was no appreciable change in *STYK1 *expression in HS832 and SkOv3 cells. In contrast to *STYK1 *RNA expression, STYK1 protein expression levels were unaffected by estradiol, BSA-conjugated estradiol, and G-1 treatments, with the possible exception of OvCar5, where a slight increase in STYK1 was observed with E2B after 16 hours post-treatment (Fig. 4).

## Discussion and Conclusion

STYK1 mRNA levels have been reported in human benign and/or malignant tissues, but the immunoreactivity of STYK1 has not been reported. Several reports demonstrate *STYK1 *mRNA expression in various normal tissues and *STYK1 *overexpression in breast and lung cancer tissues and cell lines, as well as in patients with acute leukemia [17-19]. Moreover, Moriai et. al reported high levels of *STYK1 *expression even in early stages of breast cancer. In this study, we demonstrated the presence of STYK1 immunoreactivity, in benign and malignant ovarian tissues and cell lines but not in normal ovarian tissue. Moreover, benign ovarian tissues displaying immunoreactivity for STYK1 displayed weak staining. Moderate and strong STYK1 staining was seen only in the high grade ovarian cancer tissues. This data suggests that STYK1 is associated with tumorigenic and malignant phenotypes in ovarian tissue. However, it should be noted that duplicates of only two normal tissue sections were analyzed in this study. With more samples this data should support the need for future studies to validate STYK1 as a potential prognostic tool for detecting multiple stages of ovarian carcinogenesis.

Our lab previously demonstrated that estradiol increases *STYK1 *mRNA levels in ER*α *negative, ER*β *positive MDA-MB 231 breast cancer cells [10]. In the current study estradiol downregulated *STYK1 *in OvCar5 cells expressing ERβ but not ERα but did not have a notable affect on *STYK1 *mRNA levels in OvCar8 cells, which have the same estrogen receptor expression profile (Fig. 3A). This might be due to the presence of higher levels of GPR30 downstream signaling proteins or EGFR/HER protein levels, which are involved in signaling through GPR30. This would be supported in the increase of STYK1 due to G1 treatment, OvCar5 versus OvCar8. It is notable that the level of GPR30 mRNA is not reflective of the relative levels of GPR30 protein. Further investigation into the mechanism of GPR30 expression and regulation is underway. However, the higher ERβ levels in the OvCar8 cells could account for the difference in STYK1 regulation. Interestingly, the highest *STYK1 *induction was seen in the HS832 cells (8 h, p < 0.01; 18 h, p < 0.001), which are ER*α *and ER*β *negative while the ER*α *and ER*β *positive SkOv3 cells had a marked reduction in *STYK1 *expression in response to estradiol treatment. This data suggests that there is an inverse relationship between estradiol-mediated *STYK1 *regulation and ER*α*/ER*β *expression. A similar observation was observed in MCF7, ERα positive, ERβ negative versus MDA-MB-231 which is ERα negative, ERβ positive [10]. ER*α *was previously shown to downregulate the FN1 gene in ovarian cancer cells and ERβ expression is inversely correlated with tumorigenesis in ovarian cells [11,20]. Regulation of *STYK1 *expression in cells negative for ER*α *and ER*β *points to estradiol-mediated regulation through a nontraditional hormone receptor pathway, possibility GPR30.

GPR30, a novel estrogen receptor was recently reported to mediate changes in gene expression and growth in ovarian cancer cells treated with estradiol [15]. We showed that G-1, a GPR30-specific ligand, induced *STYK1 *at a higher level in the ovarian tumor cells than estradiol. A significant increase (p < 0.001) in G-1-induced STYK1 expression was seen in OvCar5 cells, which do not express ER*α*, the primary estradiol receptor, but expresses ERβ at very weak levels. In contrast, G-1 downregulated *STYK1 *in OvCar8 cells, which expressed no ERα and the highest ERβ levels of the analyzed cell lines. We speculate that *STYK1 *is a downstream target of estrogen-mediated GPR30 activation in ovarian cancer cells and that the affect of GPR30 on *STYK1 *expression is more pronounced in the absence of ER*α *and ER*β*. This difference in *STYK1 *regulation could be due to the loss preferential or competitive binding of estradiol to ER*α *and/or ER*β*. It is important to note that one study reported that estradiol does not activate GPR30 [21]. It would also imply that the affect ER*α *and/or ER*β *on GPR30-mediated regulation of STYK1 expression occurs through a mechanism other than competitive ligand binding.

The cellular localization of GPR30 is controversial. It has been reported to localize to the cell membrane and the endoplasmic reticulum membrane [21,22]. We addressed this issue; G-1-induced *STYK1 *expression was compared to that in cells treated with BSA-conjugated estradiol (E2B), which is too large to enter the cell. Therefore, any estradiol-induced *STYK1 *expression would occur through binding of estradiol to a cell membrane receptor. E2B induced STYK1 expression at a level similar to the estradiol induction in each cell line except OvCar5, where *STYK1 *expression doubled. However, E2B-induction was consistently lower than that seen in G-1-treated cells. This data supports localization of GPR30 to the cell membrane but does not controvert reports of its intracellular localization and provides further evidence of estradiol binding to GPR30.

Liu *et. al *reported tumorigenesis and metastasis of normal cells (NIH3T3 and BaF3) overexpressing *STYK1 *in nude mice [5]. Their group suggested that abnormal expression of STYK1 results in a constitutively active state caused by disruption of an inactive vs. active state equilibrium. However, we did not see an appreciable difference in total STYK1 protein levels in cells treated with estradiol and G-1 compared to untreated controls. It is possible that changes in STYK1 protein levels occur early in tumorigenesis and that estradiol does not further induce STYK1 overexpression. Nonetheless, studies show that STYK1 activity is regulated by phosphorylation and dephosphorylation of several tyrosine residues within exon 11 [6,9]. Therefore, both STYK1 and GPR30 might be model therapeutic targets for the development of more effective ovarian cancer treatments. These molecular targets may also be especially important in treating triple negative (ER*α *negative, HER2 negative, progesterone receptor negative) breast cancers, which are often nonresponsive to standard chemotherapeutic medications that target traditional hormone receptors [10].

## Competing interests

The authors declare that they have no competing interests.

## Authors' contributions

KAJ carried out the molecular genetic studies, westerns, performed the statistical analysis, and drafted the manuscript. JH assisted and carried out the westerns. GO carried out analysis of immunohistochemistry. KSK conceived the study, and participated in its design and coordination. All authors read and approved the final manuscript.
